# Sonication-Based Improvement of the Physicochemical Properties of Guar Gum as a Potential Substrate for Modified Drug Delivery Systems

**DOI:** 10.1155/2013/985259

**Published:** 2013-08-05

**Authors:** Siddique Akber Ansari, Pietro Matricardi, Claudia Cencetti, Chiara Di Meo, Maria Carafa, Claudia Mazzuca, Antonio Palleschi, Donatella Capitani, Franco Alhaique, Tommasina Coviello

**Affiliations:** ^1^Department of Drug Chemistry and Technologies, University “La Sapienza”, 00185 Rome, Italy; ^2^Department of Sciences and Chemical Technologies, University of Rome “Tor Vergata”, 00133 Rome, Italy; ^3^Magnetic Resonance Laboratory Annalaura Segre, Institute of Chemical Methodologies, CNR Research Area of Rome, Monterotondo Stazione, 00016 Rome, Italy

## Abstract

Guar Gum is a natural polysaccharide that, due to its physicochemical properties, is extensively investigated for biomedical applications as a matrix for modified drug delivery, but it is also used in the food industry as well as in cosmetics. A commercial sample of Guar Gum was sonicated for different periods of time, and the reduction in the average molecular weight was monitored by means of viscometric measurements. At the same time, the rheological behaviour was also followed, in terms of viscoelasticity range, flow curves, and mechanical spectra. Sonicated samples were used for the preparation of gels in the presence of borate ions. The effect of borax on the new samples was investigated by recording mechanical spectra, flow curves, and visible absorption spectra of complexes with Congo Red. The anisotropic elongation, observed in previous studies with tablets of Guar Gum and borax, was remarkably reduced when the sonicated samples were used for the preparation of the gels.

## 1. Introduction

It is well known that viscometric properties of macromolecules are significantly affected by their molecular weight. The aim of this study was to examine the effect of the reduction of Guar Gum (GG) molecular weight for an appropriate modulation of its flow and gelling properties, according to specific needs and possible applications in the field of pharmaceutics, cosmetics, and food industry. Furthermore, a reduced molecular weight should allow conjugation with hydrophobic moieties which should lead to self-assembled structures suitable as systems for drug delivery and targeting as previously proposed with gellan gum, which was sonicated and chemically linked to prednisolone [1]. Furthermore, following previous investigations carried out using another polysaccharide (i.e., scleroglucan) [2], we studied the effect of borax crosslinking on the rheological properties of GG solutions sonicated for different intervals of time and the corresponding anisotropic swelling and elongation of tablets obtained by compression of the GG/borax system. The helix conformation, assumed by the GG chains in the presence of borax [3], was tested to be stable even at high pH values by means of Congo Red complexation experiments. The chosen polysaccharide, GG, is a water-soluble galactomannan which consists of a linear *β*-D-(1,4)-mannose backbone irregularly substituted by uncharged *α*-D-(1,6)-linked galactose side groups [4]. This seed gum, generally recognised as safe (GRAS), because of its gelling, viscosifying, and thickening properties is used in pharmaceutics [5] for the stabilization of emulsions and suspensions; it is also employed in tablet manufacturing as a binder and disintegrating agent and for drug micr-encapsulation. Furthermore, GG is suitable as a food supplement [6]. It has been proposed in the therapy of hypercholesterolemia, hyperglycemia, and obesity [7], but also in textile, paper, and paint industries, as well as in cosmetics. As pointed out, most practical applications of GG are related to its rheological and gelling properties; furthermore, GG is pable to form hydrogels when crosslinked with glutaraldehyde [8–11] and in the presence of borax [3]. For the reduction of GG molecular weight, the probe sonication approach was used because in such case no chemicals are needed, and such aspect represents an important prerequisite for the marketing approval of the obtained polymer.

## 2. Materials and Methods

### 2.1. Materials

GG was provided by CarboMer (USA), borax was a Carlo Erba (Italy) product, and Congo Red (CR) a Sigma Aldrich (USA) product. For the sample preparations distilled water was always used, except for NMR experiments, where deuterium oxide (D_2_O) (Cambridge Isotope Laboratories, USA) was used. All products and reagents were of analytical grade. The Polysaccharide calibration kits, SAC-10 (Agilent Technologies, UK) were used as pullulan standards.

#### 2.1.1. GG Purification

A given amount of polymer was dissolved in distilled water (polymer concentration, C_*p*_ = 0.5% w/v) and then kept under magnetic and mechanical stirring at 60°C for 24 h. The obtained solution was exhaustively dialysed at 7°C against distilled water and then freeze-dried. The molecular weight cut-off of dialysis tubing was 12,000–14,000. From now on, the sample purified by dialysis will be called GG.

#### 2.1.2. Hydrogel and Tablet Preparation

For the preparation of the tablets, an appropriate amount of polymer (about 200 mg) was magnetically stirred in water for 24 h. Then, the calculated amount (i.e., moles of borax = moles of repeating units of polymer) of 0.1 M borax solution was added, and the system was left under magnetic stirring for 5 min. The obtained sample (C_*p*_ = 0.7%, w/v) was kept overnight at 7°C for gel setting and then freeze-dried. Tablets were prepared from the freeze-dried sample with an IR die (Perkin Elmer hydraulic press) using a force of 5.0 kN for 30 s. The weight of the GG/borax tablets was 230 ± 10 mg, the diameter was 13.0 ± 0.1 mm, and the thickness was 1.05 ± 0.02 mm.

### 2.2. Methods

#### 2.2.1. Sonication

In order to reduce the GG molecular weight, a High Intensity Ultrasonic Processor (750 Watt model, probe type sonicator—Vibra Cell—VC 750, Cole-Parmer, USA) was used, working at 20 kHz, with a 6.5 mm microtip, applying an amplitude of 30% of the maximum power supplied by the instrument corresponding to 20 watts and pulser cycles of 30 s ON and 30 s OFF. Aqueous solutions (50 mL) of GG (0.5% (w/v)), prepared in glass beaker, were kept, during sonication, in an ice bath which allowed to keep an average temperature of the samples below 25°C. These experimental conditions allowed to maintain the structural characteristics of the polymeric chains as evidenced also in the case of other polysaccharides [2]. All samples, after sonication, were centrifuged for 20 min at 5,000 rpm and 20°C. The centrifuge speed was selected after several tests in order to find out the minimum rpm value capable of removing the probe-tip residues from the polymer solutions. The supernatant solutions were then dialysed and finally freeze-dried.

#### 2.2.2. Ultracentrifugation

After sonication, all samples were centrifuged (Sorvall WX 80 ULTRA centrifuge (Thermo Scientific, USA)) for 20 min at 20°C and 5,000 rpm. For an appropriate comparison, GG samples (C_*p*_ = 0.5% w/v) were only centrifuged (without applying the sonication step). All GG samples were then dialysed and freeze-dried.

From now on, the dialysed sample will be called GG while the centrifuged and dialysed samples will be called, according to the minutes of sonication, GG 0 (no sonication), GG 1, GG 3, GG 5, GG 10, GG 20, and GG 30.

#### 2.2.3. GPC

Weight molecular weight (*M*_*w*_) and polydispersity index (*M*_*w*_/*M*_*n*_) were determined by GPC on a bank of TSK gel GMPWXL columns (Tosoh Bioscience, Tokyo, Japan). A Varian 210 HPLC system with a 356-LC refractive index detector was used. The eluant was 55 mM Na_2_SO_3_ and 0.02 NaN_3_ in double distilled water; the flow rate and temperature were maintained at 0.6 mL/min and 40°C, respectively. All guar samples were diluted to 0.15% (w/v) and filtered through a 0.45 *μ*m filter prior to analysis. The bank of columns was calibrated using pullulan standards [12, 13].

#### 2.2.4. NMR Analysis

Samples of dialysed GG and GG 30, about 4 mg, were solubilized in 0.7 *μ*L of D_2_O. ^1^H-NMR experiments were carried out at 45°C on a Bruker AVANCE AQS 600 spectrometer operating at 600.13 MHz and equipped with a Bruker multinuclear, *z*-gradient probe head. A soft presaturation of the HOD residual signal was applied before spectra acquisition [14].

#### 2.2.5. Rheological Measurements

The rheological characterization of the GG and GG/borax samples was performed by means of a controlled stress Haake Rheo-Stress RS300 rotational rheometer, provided with a Haake DC50 thermostat. A cone plate device (Haake CP60Ti: diameter = 60 mm; cone = 1°; gap = 0.053 mm) was used for GG solutions while a grained plate-plate device (Haake PP35 TI: diameter = 35 mm) was employed for GG/borax samples in order to reduce the extent of wall slippage phenomena [15]. To perform the measurements, the samples were transferred in the rheometer, and the upper part of the selected device was then lowered until it reached the sample surface. Gap-setting optimizations were undertaken according to the procedure described elsewhere [16]. Stress sweep experiments were performed (25°C and 1 Hz) in the range *γ* = 0.001 ÷ 1000; frequency sweep experiments were carried out in the range 0.01 ÷ 10 Hz, in the linear viscoelastic region (usually *γ* = 0.01), preliminary assessed by stress sweep experiments; flow curves were performed in the range 0.001 ÷ 1000 s^−1^, applying a stepwise increase of the stress. All measurements were made at 25°C.

#### 2.2.6. Dilute Solutions Viscometry

For the viscosity measurements, an automatic viscometer (Instrument Schott AVS 370, Lauda, Germany) with a water bath (Lauda 0.15 T) allowing the temperature control to 0.1°C was used. An Ubbelohde capillary viscometer (Type no 531 01, with a capillary diameter = 0.54 mm, Schott-Geräte) for dilution sequences, with a flux time for the solvent (distilled water) at 25°C of 178.28 s, was used. The distilled water for the sample preparations and for the dilutions was previously filtered three times with 0.20 *μ* Sartorius Biolab Products filters. The flux time of GG solutions (*t*) was compared with that of the solvent (*t*_0_), and the relative increase of fluxing time (*t*/*t*_0_ = *η*/*η*_0_ = *η*_*rel*⁡_; *η*_*rel*⁡_ − 1 = *t*/*t*_0_ − 1 = *η*_specific_) was evaluated at different polymer concentrations. In the range of viscosities up to about twice that of water (i.e., *η*_*rel*⁡_ = 2), the following Huggins equation is valid:

(1) $$\begin{array}{c} \;\frac{\eta_{\text{specific}}}{C}=\left[\eta \right]+k_{H}{\left[\eta \right]}^{2}C. \end{array}$$

The limiting value of *η*_specific_/*C* for *C* → 0 represents the intrinsic viscosity, [*η*] (cm^3^/g), while from the slope of the linear trend of viscosity data it is possible to calculate the Huggins constant, *k*_*H*_, which gives an indication on the aggregation state of the polymer in the tested conditions of solvent and temperature [17]. The intrinsic viscosity is a measure of the inherent ability of the polymer to increase the viscosity of the solvent at a given temperature, and it can be determined, according to Huggins equation, by measuring viscosity of solutions at low concentrations and extrapolating to infinite dilution. Furthermore, intrinsic viscosity [*η*] is related to the viscosity average molecular mass of the polymer through the Mark-Houwink-Sakurada relationship (MHS), to the molar mass of the sample [18]: [*η*] = *KM*_*w*_^*a*^, where *K* and *a* are constants for each polymer-solvent system at a given temperature and are, both, related to the “stiffness” of the polymer. For flexible polymer coils, *a* is 0.5 in a *θ* solvent and 0.8 in a good solvent [12].

#### 2.2.7. Conformational Transition Studies

The capability of sonicated GG to retain the helix conformation when linked with borax [3] was examined by characterising the Congo Red-GG complexes [19, 20]. Experimentally, aliquots of a NaOH stock solution were added to GG-borax samples (1 g/L) containing 10 *μ*M CR, in order to obtain the desired pH values (9, 10, and 13). 

The solutions were left to equilibrate until their UV-Vis spectra did not change with time. For a comparison, similar experiments were carried out on a 10 *μ*M CR solution. The absorption spectra were recorded with a Cary 100 spectrophotometer (Varian, Palo alto, CA, USA) using a quartz cuvette with a path length of 1 cm. 

#### 2.2.8. Water Uptake and Dimensional Increase Studies

The swelling of GG/borax tablets, prepared with the different types of GG (i.e., dialysed GG, centrifuged and dialysed GG, and GG sonicated for different periods of time, centrifuged and then dialysed), was carried out by soaking the tablets in distilled water at 37°C. At fixed time intervals, the tablets were withdrawn, the excess of water was removed with soft filter paper for 5 s, and then the corresponding weights and dimensional variations along the longitudinal axis were determined by means of a screw gauge with an accuracy of ±0.1 mm. No remarkable variations of cross-section dimensions were detected during the swelling process. All experiments were carried out in triplicate, and the obtained values always lay within 10% of the mean.

## 3. Results and Discussion

### 3.1. GPC Measurements

**Figure 1:**
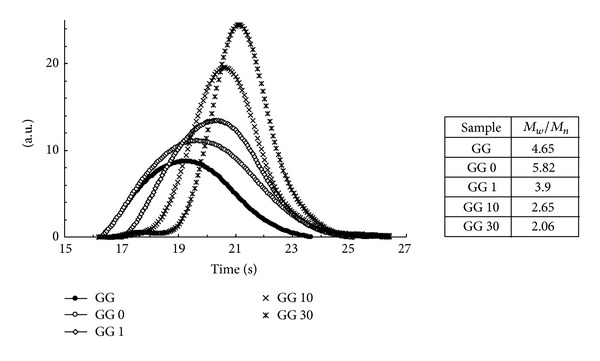
Molar mass distributions of the guar samples and table of Polydispersity Index.

Molecular weight distributions of GG and sonicated GG samples by GPC are shown in Figure 1. It is apparent that depolymerization of GG by sonic irradiation causes degradation of the polysaccharide to give different fractions of lower molecular weight, each fraction having a narrow molecular weight distribution. In absence of external stimuli (GG and GG 0), the distribution curves are very broad while by applying sonication for different period of time, the peaks become sharper. The molecular weight distribution is in fact somehow more important for characterising the samples than just their *M*_*w*_ only. In Figure 1, typical chromatograms of GG samples are shown, together with the polydispersity index (*M*_*w*_/*M*_*n*_). It should be kept in mind that the sonicated samples are also centrifuged (and finally dialysed), and this procedure removes not only the tip dust but also the possible aggregates present in the samples. On the other hand, in the dialysed sample (GG), only the polymeric fractions of very small molecular weights (cut-off of dialysis tubing: 12.000–14.000) and impurities, if present, are removed. Thus, the dialysed samples lack the smallest molecular weight fractions while the sonicated ones lack also molecular aggregates, if present. The high polydispersity index detected for GG 0 sample may be due to the effect of centrifugation that breaks the aggregates leading to the dissolution of lower molecular weight fractions (though higher than 14.000); such effect obviously does not occur in the sample that was only dialysed. From the table of Figure 1, it is evident how the sonication process reduces significantly the polydispersity index leading for an irradiation of 30 min, towards the value expected for the most probable distribution in a random chain scission (*M*_*w*_/*M*_*n*_ = 2).

### 3.2. Rheological Measurements

**Figure 2:**
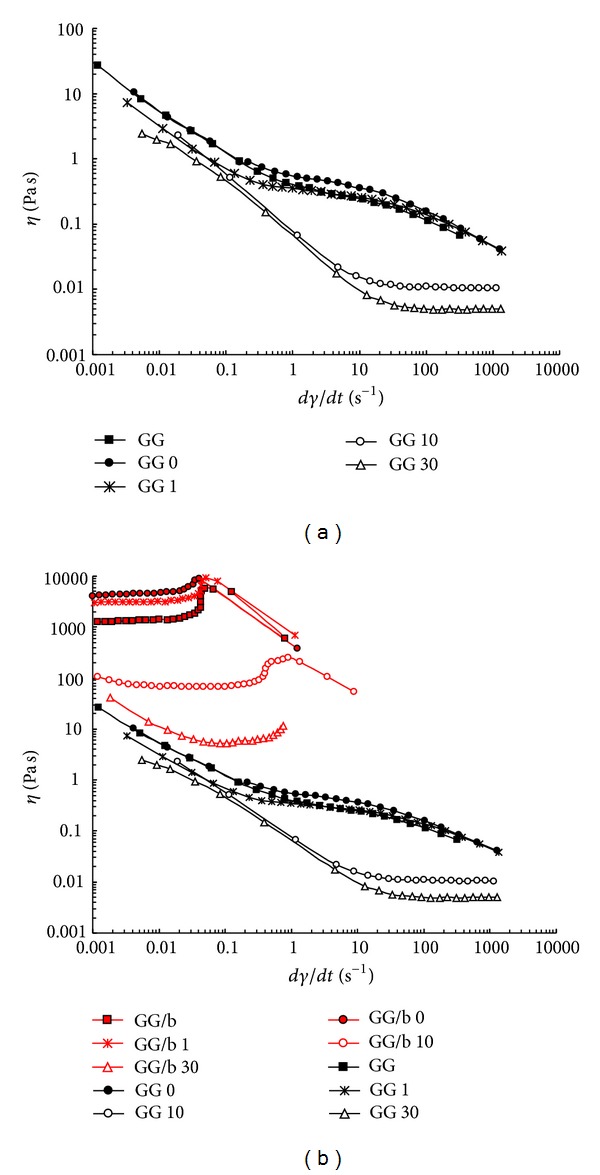
(a) Flow curves of GG samples sonicated (at C_*p*_ = 0.5%) for different periods of time (0, 1, 10, and 30 min). (b) Flow curves of GG samples, with (red symbols) and without borax (black symbols): for a comparison, the flow curves recorded for GG and GG 0 are also reported (C_*p*_ = 0.7%, *T* = 25°C).

The flow curves for the sonicated samples are shown in Figure 2. As a comparison, the flow curves of GG and GG 0 are also reported. It can be noted that these two samples, together with GG 1, show almost superimposable profiles. The small differences in the three profiles can be attributed to the different molecular weight distributions, as evidenced by the GPC measurements. The first Newtonian plateau is not detectable for any sample, and the drop of viscosity is quite significant until the second Newtonian plateau is reached with values of viscosity approximately less than 1 Pa s. By increasing the sonication time, drastic changes are observed in the flow curves. A sonication of ten min is sufficient to drastically reduce the viscosity of the polymer (at *dγ*/*dt* = 10 (s^−1^)) which drops of one decade, from 0.3 (for GG and GG 1) to 0.02 (Pa s). Furthermore, the interval of the Newtonian plateau is remarkably shifted to a higher shear rates indicating that, by reducing the chain length, the viscosity continues to decrease while, for the native polymer or the polymer sonicated for 1 min, the viscosity values remain almost constant up to *dγ*/*dt* = 100 (s^−1^)). Thus, ten minutes of sonication are sufficient to break significantly the GG chain backbones (the initial molecular weight was reduced to one third, see Section 3.3). Furthermore, by increasing the period of sonication, the range of the second Newtonian plateau is even more shifted towards higher shear rate values, and the macroscopic effect of a sonication of 30 min is that the viscosity approaches values only five times higher than those of water. 

**Figure 3:**
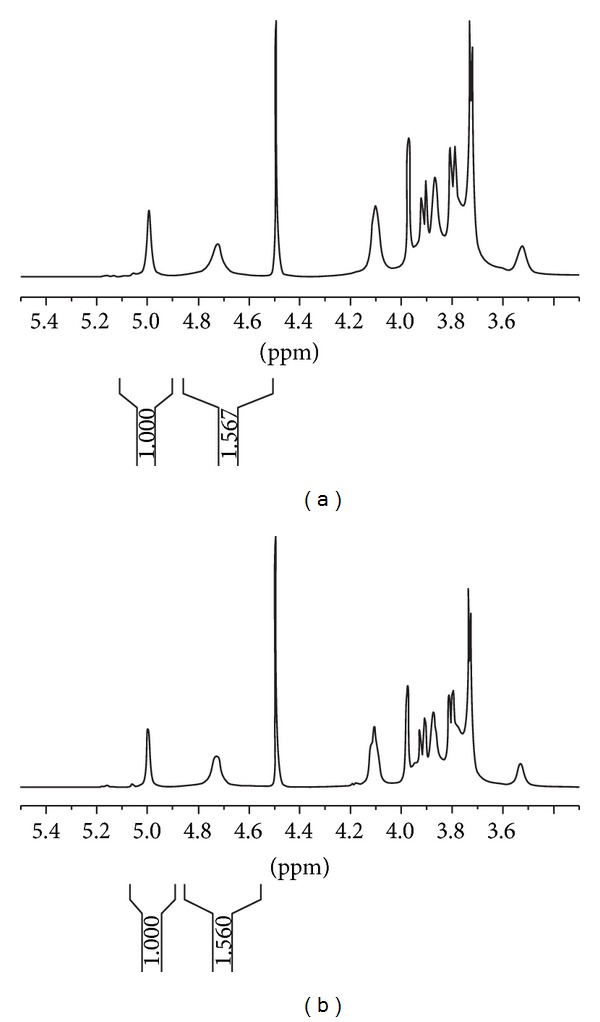
^1^H-NMR spectra at 600.13 MHz and at 45°C (D_2_O) of dialysed GG (a) and GG 30 (b).

Increasing the sonication time, a further breakdown of the polymeric chains occurs without damaging the repeating unit structure, as evidenced by NMR analysis (Figure 3).

NMR was carried out in D_2_O at 45°C, in order to separate the HOD residual signal from the anomeric signals. As shown in Figure 2, the spectra of GG and GG 30 are superimposable. In particular, the ratio between the anomeric *β* (1→4) mannose protons at 4.73 ppm and the anomeric *α* (1→6) galactose protons at 5.02 ppm remains constant and equal to 1.56 for the tested samples. This is a clear indication that the *M*/*G* ratio is not affected by the sonication process; that is, the repeating units of the polymer keep intact their chemical compositions.

Thus, the sonication process can be proposed as a suitable method for GG molecular weight reduction. Actually, by means of sonication, it is possible to prepare GG within a quite wide range of molecular weights without damaging the molecular structure. This is a crucial point, especially with reference to possible industrial applications of GG, because the rheological properties of the polymeric solutions prepared with the sonicated samples that show remarkable variations in comparison with the native polymer, can be appropriately tailored according to specific needs.

The effect on the rheological properties of the addition of borax to the various samples was also investigated (Figure 2). Addition of borate ions to the GG samples led to a dramatic change in the flow curve trends. Between the samples without borax and those with borax, a difference in the viscosity values of four decades is detected. A well-defined plateau is recorded at low shears, up to *dγ*/*dt* = 0.1 (s^−1^). After this value, GG and GG 0 undergo a rapid decrease in viscosity followed by the impossibility to further proceed with the measurements due to the brittle nature of the network. The presence of borax leads to a better discrimination among the different samples even after 1 min of sonication. Going from 1 to 10 min of sonication, the viscosity is strongly reduced and the plateau is extended up to *dγ*/*dt* ≈ 2 (s^−1^). Thus, the reduction in molecular weight induces a dramatic change in the polymeric network. When sonication is carried out for 30 min, the sample with borax starts to show a pseudoplastic behaviour. However, in no case the viscosities of the GG/b samples reach values below those recorded for the samples without borax. It is clear that both GG molecular weight and borax represent key parameters for the flow properties.

**Figure 4:**
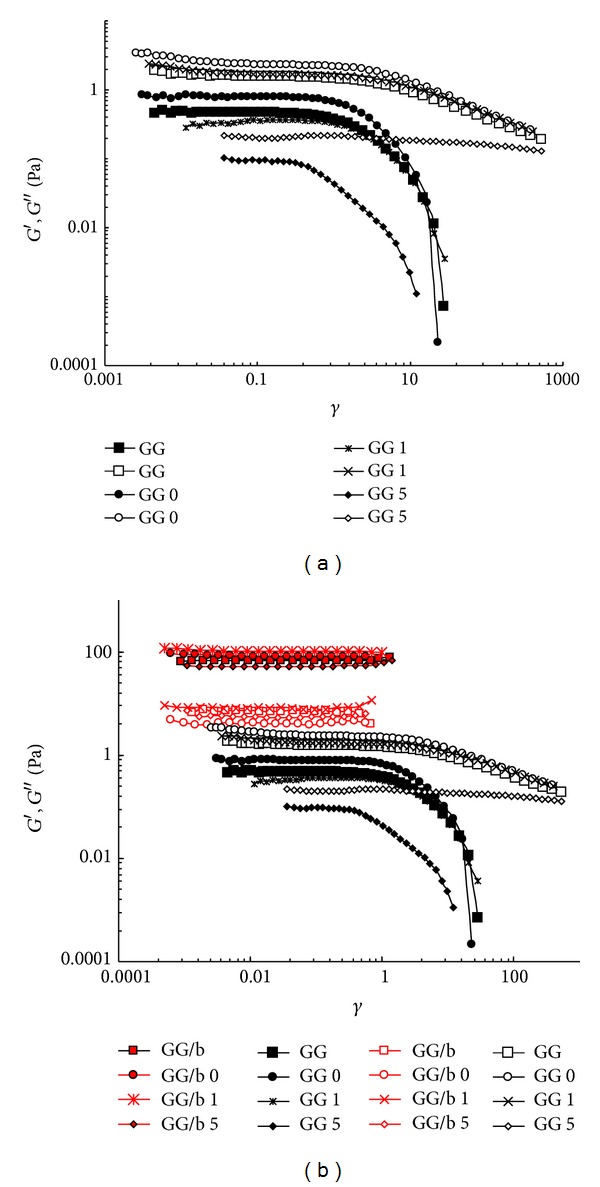
(a) Viscoelasticity plot of GG samples sonicated (at C_*p*_ = 0.5%) for different periods of time (0, 1, and 5 min). (b) Viscoelasticity curves of GG samples, with (red symbols) and without borax (black symbols): for comparison, the flow curves recorded for GG are also reported (*G*′ = full symbols, *G*′′ = empty symbols; C_*p*_ = 0.7%, *T* = 25°C).

In Figure 4, the changes of the two moduli, *G*′ (storage modulus) and *G*′′ (loss modulus), as a function of the deformation are shown. As expected, the linear viscoelasticity interval is reduced by lowering the sample molecular weight. The addition of borax increases the *G*′ values from about 1 to approximately 100 Pa and the stress/strain response is linear up to a 100% of deformation. The mechanical spectra were then recorded at *γ* = 0.01. 

**Figure 5:**
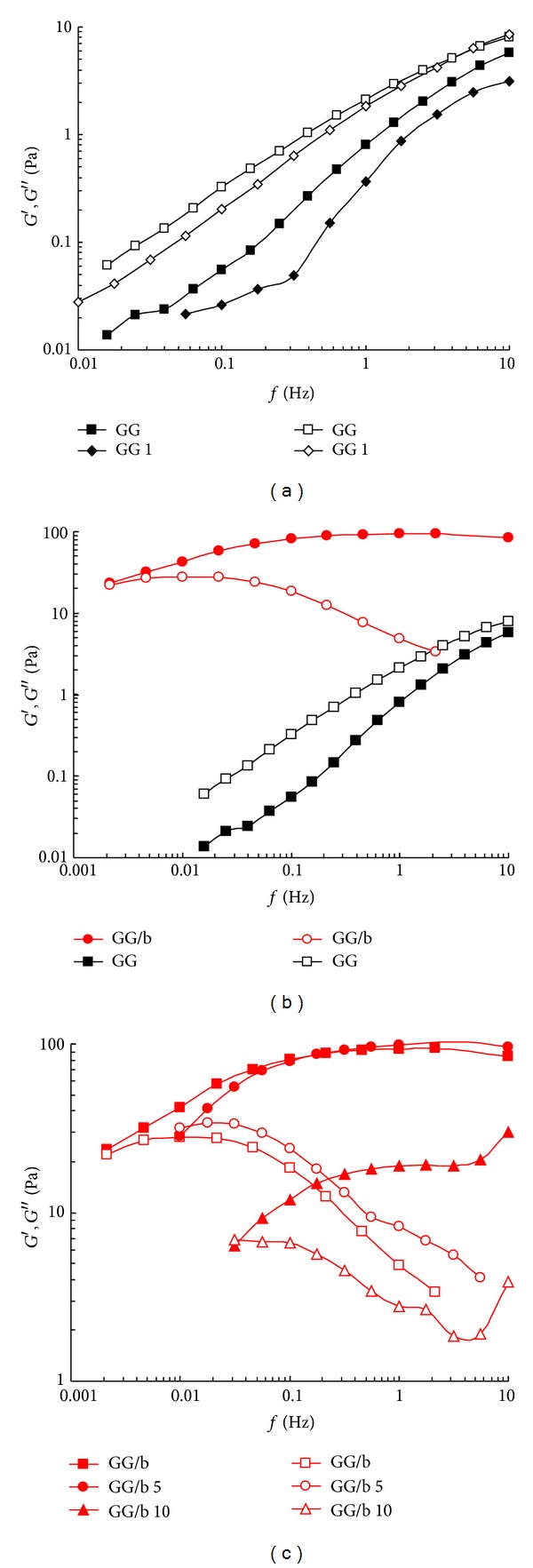
Mechanical spectra of GG samples, with (red symbols) and without borax (black symbols), sonicated (at C_*p*_ = 0.5%) for different periods of time. (a) Frequency sweep of GG and GG 1; (b) frequency sweep of GG and GG/b; (c) frequency sweep of GG/b and GG/b sonicated for 5 and 10 min; (c) comparison between the frequency sweep of GG and GG/b. (*G*′ = full symbols, *G*′′ = empty symbols; C_*p*_ = 0.7%, *T* = 25°C).

In Figure 5, the mechanical spectra of GG samples are reported. It is interesting to follow the modulus variations in the stress sweep experiments, which show the significant effect of sonication on the supramolecular structure.

In Figure 5(a), the effect on GG sample of 1 min sonication is reported: both mechanical spectra are those typical of a solution (*G*′′ > *G*′), and a strong dependence of the two moduli on frequency can be detected. In the case of samples sonicated for a longer period of time, it was not possible to carry out the experiment, due to their predominant liquid-like characteristics.  Figure 5(b) compares the dynamic rheological moduli, prior to degradation, of a GG solution with a GG-borax gel at the same polymer concentration (0.7% w/v). There is a dramatic effect on the shape of frequency sweep trend as a consequence of the addition of borax: a well-defined transition from a liquid-like to a gel-like behaviour is detected. Actually, the loss modulus (*G*′′) dominates the solution response over most of the frequency domain, indicating the mainly viscous nature of the GG solution. Borax crosslinking causes an increase in both moduli, with the elastic modulus (*G*′) being more strongly affected. A well-defined plateau is observed in the elastic modulus at intermediate frequencies (0.05–10 Hz), being *G*′ higher than *G*′′ within this interval. These features are characteristic of a network structure formed by the GG-borax crosslinks. Due to the labile nature of these linkages, the GG/b gel displays a terminal region similar to that of polymer solutions with a well-defined crossover between *G*′ and *G*′′. The frequency values at which the moduli inversion takes place increase as sonication time increases. Obviously, by reducing the GG molecular weight, the strength of the gel, formed in the presence of borax, correspondingly decreases. This is clearly evidenced by the reduction of the storage modulus values of the frequency interval where *G*′ is independent of the applied frequencies and of the shift of the crossover towards higher frequency values. All these variations indicate the increasing weakness of the polymeric network as sonication proceeds with a widening of the mesh size (with the reduction of *G*′ values) and a corresponding shortening of the longest relaxation time (the crossover frequency is shifted towards higher values).

In Figure 5(c), the effects of sonication on GG/b samples are evidenced. The addition of borax to GG 5 leads to a network very similar to that of GG/b. Nevertheless, it must be pointed out that the crossover between *G*′ and *G*′′ is appreciably shifted to higher frequencies in comparison to GG/b sample (from 0.002 Hz (GG/b) to 0.012 Hz (GG/b 5). In the case of sonicated samples, the sol-gel transition occurs at higher frequencies due to the reduced influence of the longest relaxation modes, as expected for a looser network. Furthermore, the maximum of *G*′′ does not match with the crossing point: this discrepancy is reduced by increasing the sonication time. This means that, by reducing the chain length, the data can be fitted by using the generalized Maxwell model applying only a few or only one (in the case of coincidence between the *G*′′ maximum and the crossing point) relaxation time. This is even more evident when GG undergoes a 10 min sonication. The mechanical spectra are still those of a gel system, but both moduli are lowered by a power of ten, and the crossover is further shifted to higher frequencies (*≈*0.3 Hz). Of course, in this last case a much weaker gel is obtained, being the *G*′ plateau significantly reduced in comparison to that of the previous two systems. 

Anyhow, in the case of GG, one min is the maximum sonication time that can be applied in order to record the mechanical spectra (a more prolonged sonication leads to liquid-like systems with moduli that are too low to be experimentally recorded). On the other side, the presence of borax, leading to the formation of a three-dimensional network, allowed to acquire mechanical spectra of a GG sample sonicated up to 10 min. When the sonication time is further increased to 30 min the linkages with borax are too few, due to the reduction of GG molecular weight (*M*_*w*_ = 1.32 × 10^5^) for the formation of a real gel network, and consequently the modulus values become too low to be recorded. 

### 3.3. Reduction of Guar Gum Solution Viscosity upon Sonication

**Figure 6:**
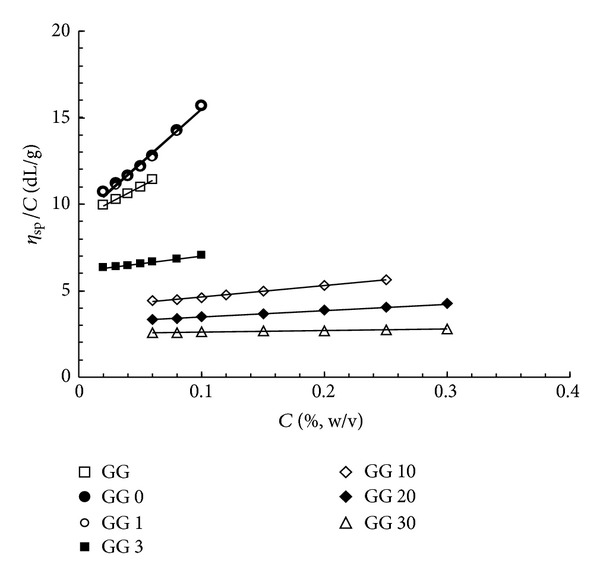
(a) *η*_sp_/*C* versus polymer concentration for GG samples sonicated for different periods of time (*T* = 25°C).

Aqueous solutions of sonicated samples were prepared for the viscosity measurements, carried out at 25°C, and the results are shown in Figure 6. For an appropriate comparison, GG samples were also tested.

From the intercepts, that is, from the intrinsic viscosities [*η*] of the samples, the molecular weights of the various fractions were estimated (and reported in Table 1) according to the Mark-Houwink-Sakurada (MHS) equations valid for galactomannans [21]:

(2) $$\begin{array}{c} \left[\eta \right]=KM_{w}^{a}, \end{array}$$

where *K* = 5.13 × 10^−4^ and *a* = 0.72.

It is possible to observe that, by applying the sonication for only 5 min, the GG molecular weight was reduced to more than one half. The reduction was further increased by increasing the sonication process for longer times. From the slope of the linear trend of viscosity data, the values of the Huggins constant, *k*_*H*_, (see Table 1) were calculated for all samples. The Huggins coefficient is a measure of polymer-polymer interactions in solution (with values of roughly 0.30–0.40 in good solvents and 0.50–0.80 in *θ* solvents) and assumes high values when intermolecular associations exist [22]. Furthermore, it is known that water is a fairly good solvent for GG. However, *k*_*H*_ of GG in water is much higher than the regular value for a good solvent (0.3–0.4), which indicates the importance of polymer-polymer hydrogen bonding interactions among the GG molecules. When sonication is applied, the decrease in molecular weight reduces the number per chain of hydrogen bonding sites leading, at the same time, to a reduction of the intermolecular hydrogen bonding between polymer molecules, that is, the attractions between guar chains [12]. *k*_*H*_ is also very sensitive to the formation of molecular aggregates. In other words, the Huggins coefficient can provide additional information about the state of guar gum macromolecules in solution and can be a measure of the goodness of the solvent [23].

### 3.4. Water Uptake and Dimensional Increase Studies

**Figure 7:**
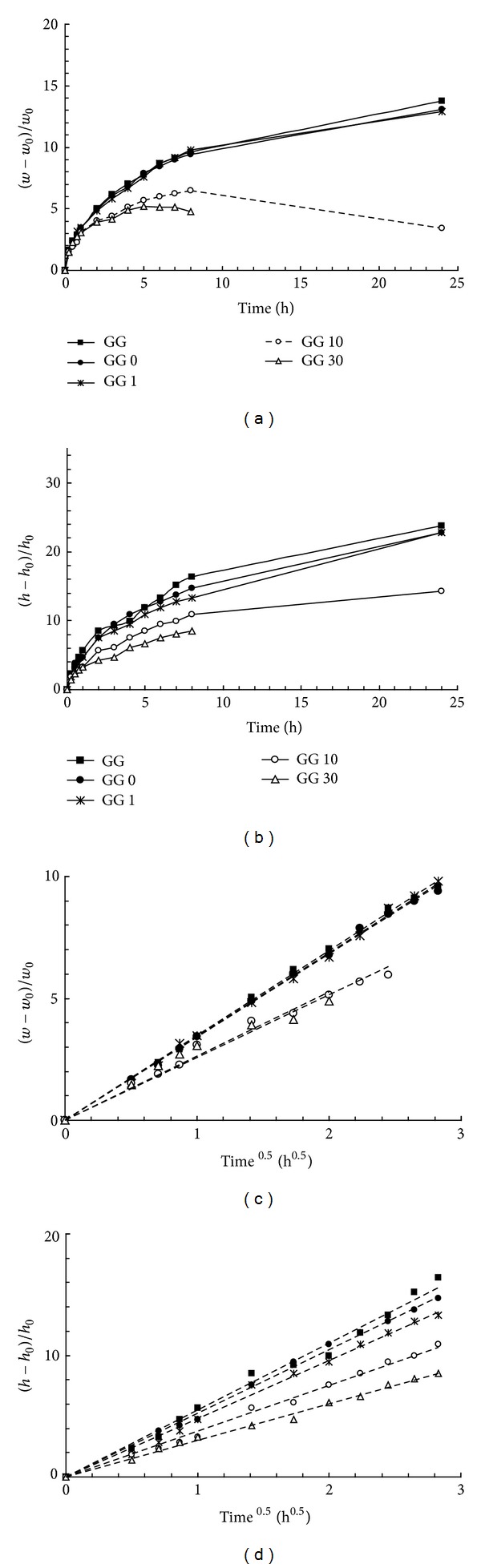
(a) Water uptake (*w* − *w*_0_)/*w*_0_ and (b) relative height increase (*h* − *h*_0_)/*h*_0_ from tablets of GG/borax (using GG samples sonicated for different periods of time) as a function of time. (c) and (d) the same data of (a) and (b) reported as a function of the square root of time. Experiments were carried out in triplicate, and the obtained values always lay within 10% of the mean.

In previous works [24], it was reported that tablets, prepared with the GG/borax freeze-dried hydrogel, undergo an anomalous peculiar swelling, essentially along one direction and very similar to that reported for the Scleroglucan/borax tablets [1, 25–27]. New tablets were then prepared with sonicated GG samples, and water uptake and elongation behaviour were followed as a function of time. As a comparison, the experiments on tablets prepared with GG (i.e., with the polymer that was only dialysed) were also carried out. The results, reported in Figure 7 (in distilled water at 37°C), clearly show that both, water uptake and anisotropic elongation of the tablets, observed for the GG/borax system, were significantly reduced when the sonicated samples were tested.

The tablets prepared with GG and GG 0 increased their weight and elongated for 24 hrs; those prepared with GG sonicated for 1 min follow the same trend while those prepared with GG 10 started to dissolve in the medium already after 8 hours. This phenomenon started even earlier for the sample sonicated for 30 min: the tablets started to loose their weight already after 5 hours, and the anomalous elongation could be detected only up to 5 hours. After 8 hours, the tablets dissolved completely in the swelling medium. Both processes, the uptake of water and the height increase, obey, during the first hours, the Fickian law (Figures 7(c) and 7(d)). Thus, the reduction of the polymeric chain lengths did not change the diffusional character of water molecules behaviour during the imbibition of the tablet matrices.

**Figure 8:**
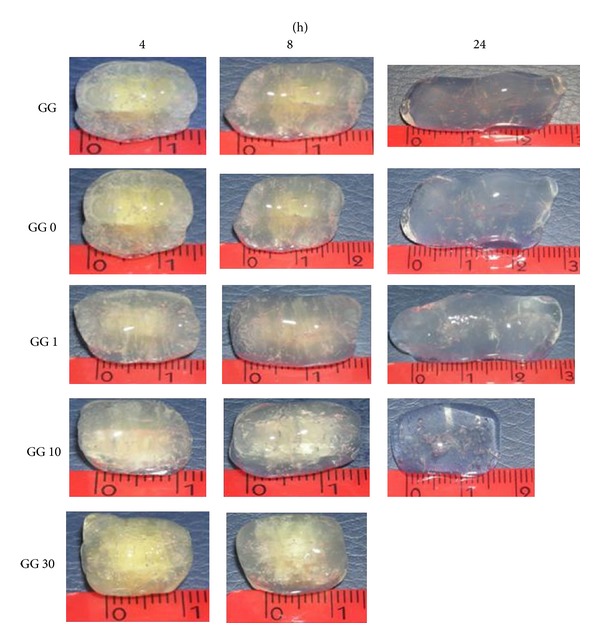
Pictures of swelled GG/borax tablets, prepared with GG sonicated for different periods of time in distilled water at 37°C (the height of the tablets before the swelling process was *h*_0_ ≈ 1.05 mm).

In Figure 8, the pictures of swelled tablets prepared with GG sonicated for different periods of time are shown. 

### 3.5. Helix-Coil Transition Analysis

**Figure 9:**
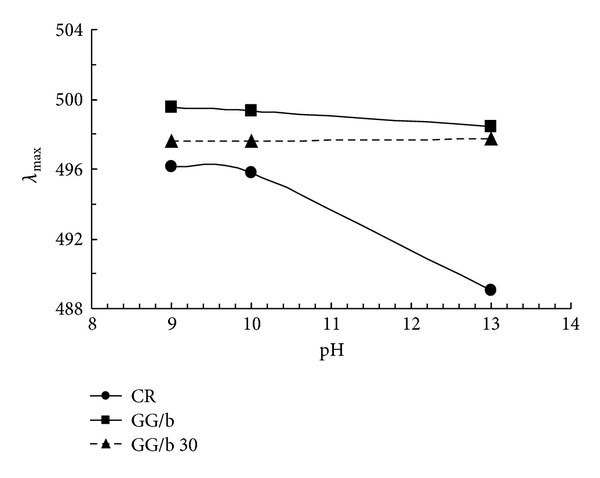
Absorption maximum shifts, at different pH values, for CR and GG/b systems with GG sonicated 0 and 30 min.

The conformational structures of GG-borax and sonicated GG/borax were investigated by means of the formation of CR-polymer complexes at various alkali concentrations. CR, indeed, tends to bind to polysaccharides in helical structures, and as a consequence, its visible absorption maximum shifts to higher values. By following its shift in the Vis spectra of the polymer-dye complex, it is possible to demonstrate the presence of helix conformation. As reported in Figure 9, the CR absorption maxima of GG/borax and GG/borax sonicated samples are red shifted in comparison to those of the dye alone, even at high pH values, indicating that the polymer at low molecular weight is still able to bind the dye, keeping a helix structure.

## 4. Conclusions 

Sonication appears to be a rather easy method suitable to reduce the *M*_*w*_ of a polysaccharide such as GG without the use of chemicals (i.e., without producing side products that could contaminate the final product) thus allowing more easily acceptable applications in the field of pharmaceutics, cosmetics, and food industry. As evidenced in the NMR spectra, sonication does not destroy the structural characteristics of the polymeric chains; furthermore, obtained results showed that depolymerization of GG by sonic irradiation leads to different fractions of lower *M*_*w*_, each fraction having a narrow molecular weight distribution. As expected, such *M*_*w*_ reduction dramatically affects the rheological properties of the polysaccharide, leading to significantly modified flow curves. In this sense, it must be pointed out that it was possible to acquire the mechanical spectra only for the sample sonicated for 1 min because the moduli of the other samples, obtained after sonication was carried out for a longer period of time, were too low to be detected. As in the case of other polysaccharides (3), the addition of borax changed completely the rheological properties from liquid-like systems to gel-like systems, and this behaviour, although to a less extent, was observed for all sonicated samples. Only when the molecular weight was decreased to *M*_*w*_ = 1.32 × 10^5^, the GG chains were no more able to build up a three-dimensional gelled network, and the rheological moduli were too low to be experimentally detected. Actually, the GG molecular weight, as estimated by viscometric measurements, decreased very rapidly by applying ultrasounds to the polymeric solutions, leading to a reduction to one-half of the initial *M*_*w*_ already after a sonication of 3 minutes.

The swelling behaviour of GG/borax tablets was also investigated. In this respect, the reduced molecular weight of the polymer led to a significant difference in water uptake and anisotropic elongation in comparison to the starting GG sample.

In conclusion, the use of a simple method, such as sonication, for the reduction of GG *M*_*w*_, represents an important approach for an appropriate tailoring of the mechanical properties of GG solutions in consideration of a more suitable and rational utilization of such polysaccharide in the various and wide fields of practical uses. In particular, as far as innovative drug formulations are concerned, an optimized reduction of the GG *M*_*w*_ will allow an easy conjugation of the obtained shorter chains with a hydrophobic drug that should then lead to the formation of self-assembled structures, in the form of nanohydrogels, that are suitable for modified drug delivery systems, as already successfully carried out and reported in the case of sonicated gellan gum chemically linked to prednisolone [1].

## Figures and Tables

**Table 1:** Sonication times of the GG samples, the corresponding intrinsic viscosity, the Huggins constant and the average molecular weight calculated according to the MHS equation.

Sample	Sonication time (min)	[*η*] (dL/g)	*k*_*H*_	*M*_*w*_ × 10^−5^
GG	0	9.15	0.44	8.02
GG 0	0	9.23	0.74	8.13
GG 1	1	9.14	0.75	8.01
GG 3	3	6.13	0.43	4.60
GG 5	5	5.24	0.49	3.70
GG 10	10	3.97	0.42	2.52
GG 20	20	3.10	0.39	1.78
GG 30	30	2.50	0.15	1.32
